# The contribution of the koniocellular visual pathway to aversive learning in human visual cortex

**DOI:** 10.1152/jn.00288.2025

**Published:** 2026-05-11

**Authors:** Katherine J. McCain, Nathan M. Petro, Andreas Keil

**Affiliations:** 1 Department of Psychology, University of Florida, Gainesville, Florida, United States; 2 Institute for Human Neuroscience, Boys Town National Research Hospital, Boys Town, Nebraska, United States

**Keywords:** differential aversive conditioning, koniocellular visual pathway, luminance channels, ssVEP

## Abstract

The present study examined the extent to which koniocellular inputs to the visual cortex are sensitive to aversive conditioning, as indexed by electrocortical activation modulated by conditioned threat. A simple differential aversive conditioning task was used to evoke visuocortical activation in response to conditioned threat (CS+) and safety (CS−) cues in each condition (tritan and luminance). The tritanopic technique was used to convey cues to S-cones that transmit color-opponent signals to regions along the koniocellular visual pathway (tritan condition). In addition, a luminance condition was included to examine visuocortical activation in response to achromatic cues that preferentially activate luminance channels (luminance condition). Steady-state visual evoked potential (ssVEP) responses to conditioned threat and safety cues were analyzed in each condition (luminance and tritan) using a nonparametric Bayesian bootstrap approach. Results showed decisive evidence (logarithmic Bayes factors, logBF10 > 2) of aversive learning in the tritan and luminance conditions, indicated by increased ssVEP envelope amplitudes in response to the CS+ compared to the CS− stimuli in occipital sensors. To further examine the koniocellular involvement in aversive learning, transitive Bayes factors were computed to compare the relative magnitude of the conditioning effects across conditions. Although transitive Bayesian comparisons showed decisive evidence indicating the magnitude of the luminance conditioning effect was larger than the tritan conditioning effect, differences in adaptation across conditions warrant further investigation. Importantly, the present study demonstrated that both koniocellular and luminance channel inputs were modulated by aversive conditioning in the human visual cortex.

## NEW & NOTEWORTHY

Previous research into the cortical representation of visual information modulated by aversive conditioning has shown that magnocellular and parvocellular inputs to primary visual cortex are sensitive to aversive outcomes. However, the role of the koniocellular pathway in mediating aversive learning in the visual cortex has yet to be explored. Crucially, the present study provides robust evidence of the koniocellular involvement in the cortical representation of conditioned threat, extending our functional understanding of the koniocellular pathway.

## Graphical Abstract

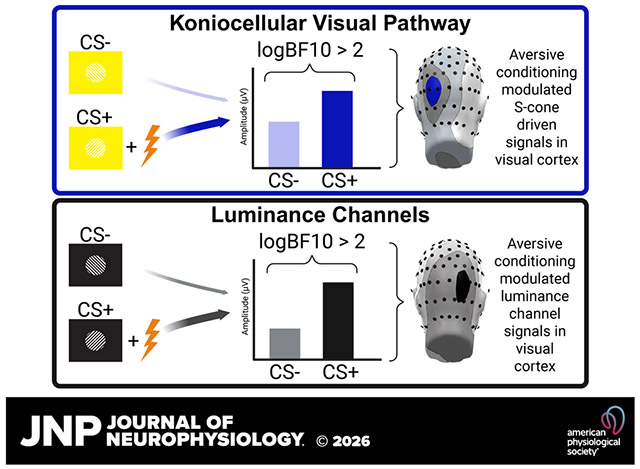

## INTRODUCTION

The human visual cortex is capable of acquiring conditioned threat and safety contingencies through differential aversive conditioning (1-3). Some research has shown that this process is driven by early visual pathways, each specialized to transmit specific visual information from the sensory environment to the visual cortex (4, 5). These investigations focused on the magnocellular and parvocellular early visual pathways, which contribute to luminance channels (6, 7), and demonstrated their involvement in the representation of conditioned threat and safety cues in visual cortex (1-3). Neural re-tuning in response to aversive cues was evident in the visuocortical enhancement of responses to conditioned threat relative to conditioned safety cues (8, 9). Thus, the role of luminance channels in mediating aversive learning in the visual cortex is well established, but the involvement of the koniocellular visual pathway has yet to be examined. Therefore, the present study used a simple differential aversive conditioning task to examine the degree to which the koniocellular visual pathway is sensitive to aversive conditioning. The tritanopic technique was used to preferentially activate the color-opponent response properties of the koniocellular pathway (10). Blue-on yellow-off signals can arise from short-wavelength cone (S-cone) stimulation while adapting the medium- (M) and long-wavelength (L) cones using the tritanopic technique (10). This technique has been used to isolate the koniocellular visual pathway involvement in motion processing (11). Furthermore, in addition to biasing the color-opponent response properties of the koniocellular pathway, luminance channel-mediated responses were also examined using achromatic stimuli.

Support for the notion that the koniocellular visual pathway may contribute to aversive learning in visual cortex stems from a vast body of research showing evidence of the cortical amplification of S-cone signals. Functional investigations have utilized S-cone biasing stimuli and observed cortical amplification of the S-cone signals at the level of primary visual cortex (12-16). For instance, Mullen et al. (12) identified a differential increase in blood-oxygenation-level-dependent (BOLD) activation in response to S-cone biasing stimuli in humans: S-cone biasing stimuli prompted small BOLD responses at the level of the lateral geniculate nucleus (LGN), but strong responses in V1. Based on this evidence, the koniocellular pathway is involved in the amplification of S-cone opponent signals, and the enhancement of these signals occurs at the cortical level.

In addition, electrophysiological investigations have also shown evidence of robust visuocortical responses when S-cones were preferentially activated (17-19). However, the extent to which attentional processes modulate visuocortical amplification of S-cone biasing signals is less understood. Comparisons across visual-evoked potential responses toward cued and uncued S-cone biasing stimuli resulted in an absence of attentional modulation (20, 21). In addition, evidence of learned aversive associations between chromatic visual stimuli and aversive unconditioned stimuli has not been readily observed (5, 22). For instance, visuocortical responses to color-opponent Gabor patches did not result in electrocortical (22) and showed limited functional (5) evidence of aversive learning. In contrast, Martinovic et al. (23) observed attentional modulation of the N1 early sensory event-related potential component during a coherent motion detection task utilizing S-cone biasing stimuli.

Additional support for the hypothesis that the koniocellular pathway plays a role in the modulation of visuocortical activation is derived from evidence of neurophysiological connections to subcortical and extrastriate regions in primates that are thought to be involved in the detection and evaluation of salient visual information (24-28). The superior colliculus, involved in early encoding of salient visual information and the execution of ocular movements (29, 30), exhibits afferents that project to the koniocellular layers of the LGN in primates (24, 25). In addition, the koniocellular layers of the LGN exhibit a direct projection to the motion sensitive middle temporal (MT) region of extrastriate cortex in primates (26). Findings in humans suggest that the koniocellular pathway is involved in fast saccadic responses to complex biologically relevant S-cone biasing stimuli (31). Similarly, it has been established that the koniocellular pathway is sensitive to motion and exhibits functional and electrocortical evidence of responses to moving S-cone biasing stimuli (11, 15).

The present study used differential aversive conditioning and the steady-state visual evoked potential (ssVEP) technique to continuously measure visuocortical responses to conditioned threat (CS+) and safety (CS−) cues. Conditioned cues were conveyed to luminance channels through achromatic, high contrast stimulation and to the koniocellular pathway by preferentially activating blue-on yellow-off color-opponent response properties (tritan stimulation). Using a previously described technique, near-equiluminant tritan stimuli functioned to predominantly activate S-cones (10) while mitigating minor S-cone input to luminance channels (32-35). Differential aversive conditioning involves two distinct stimuli where one is reliably paired (CS−) with an aversive unconditioned stimulus (US) and the second is never paired (CS−) with the US (3, 36). The inclusion of both conditioned safety (CS−) and threat cues (CS+) allowed participants to learn to discriminate threat from safety cues. Evidence of aversive learning was measured by differencing the steady-state responses to the threat and safety cues within each condition, eliminating effects of general arousal. The ssVEP technique facilitated the measurement of visuocortical responses that oscillate at the same frequency as the visual stimulation, providing robust, reliable indices of visuocortical engagement with task cues (37, 38).

Leveraging the ssVEP technique and the response properties of early visual pathways allowed for the quantification of the degree to which the koniocellular pathway and luminance channels contribute to aversive learning in visual cortex, separately. We expected the tritan stimuli to elicit robust electrocortical responses, consistent with several studies that observed S-cone signal amplification in visual cortex (17-21). We also expected the conditioned visuocortical responses to S-cone biasing stimuli to mirror those of the responses conveyed by the luminance channels, given the reliably observed visuocortical enhancement of S-cone signals. Therefore, we expected greater ssVEP amplitude in response to the conditioned threat (CS+) cues, compared to the conditioned safety (CS−) cues in both the tritan and luminance conditions (1, 2). As a manipulation check, subjective expectancy ratings (likelihood of receiving US in either of the CS stimuli conditions) were hypothesized to show further evidence of differentiation between conditioned threat and safety cues, with higher threat expectancy for the CS+ stimuli compared to the CS− stimuli after conditioning. As an additional validation metric, we measured pupil dilation, expecting greater re-dilation for the CS+ compared to the CS− stimuli in each condition because pupil re-dilation is an index of emotional arousal and sympathetic activation (39).

The present investigation quantified the level of support for our main hypotheses using a nonparametric Bayesian bootstrap approach (40, 41). Bayesian analyses allowed for the examination of the sensitivity of ssVEP responses evoked by S-cone biasing and achromatic stimuli to differential aversive conditioning in the tritan and luminance conditions, respectively. To further elucidate the involvement of the koniocellular pathway in the cortical representation of conditioned threat cues, transitive Bayesian comparisons were also conducted. Comparisons explored the relative extent to which conditioning effects observed within conditions (luminance and tritan) remained robust when compared across conditions.

## MATERIALS AND METHODS

### Participants

Fifty (means ± SD, 19.46 ± 1.95 yr; 36 females) undergraduate students were recruited at the University of Florida in exchange for course credit. All participants provided written informed consent and reported normal or corrected-to-normal vision and a negative personal and family history of seizures. All study procedures were in accordance with the Declaration of Helsinki and approved by the University of Florida Institutional Review Board.

### Experimental Design

#### Stimuli.

**Figure 1.**
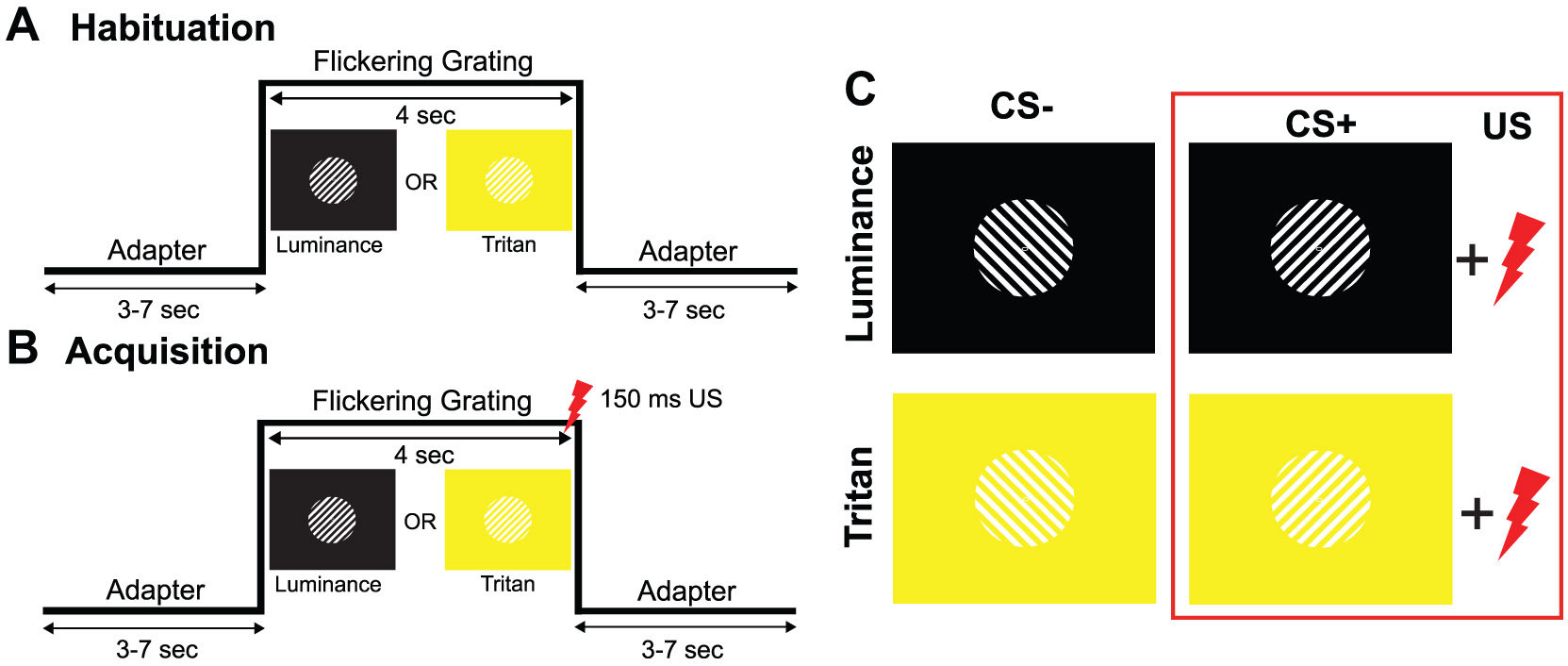
The differential aversive conditioning task consisted of luminance and tritan conditions. Trials involved adapter periods (3–7 s) that flanked the flickering grating (7.5 Hz) period (4 s). The adapter preceding the flickering grating was always a black background in the luminance condition and a bright yellow background in the tritan condition. *A*: in the habituation phase, the flickering grating was presented without any unconditioned stimulus (US) (mild electrical stimulus) pairings. *B*: in the acquisition phase, the orientation (45° or 135°) of the flickering grating signaled the presence (CS+)or absence (CS−) of the US (150 ms) and was counterbalanced across participants. *C*: conditioned stimulus (CS) exemplars for the luminance and tritan conditions. In the acquisition phase, the CS− was never paired with the unconditioned stimulus (US; mild electrical stimulus), but the CS+ was always paired with the US.

Visual stimuli were developed in MATLAB (MathWorks) using Psychophysics Toolbox version 3.0.15 (42) and presented on a Cambridge Research Systems Display + + monitor (1,920 × 1,080 pixels, 120 Hz refresh rate) connected to a PC running Linux Ubuntu. Each trial began with an adapter prestimulus period (3–7 s) that consisted of a fixation dot (visual angle 0.19°) in the center of a bright yellow background (190, 190, 0 RBG; 55.30 cd/m^2^) in tritan conditions or a black background (0, 0, 0 RBG; 0.05 cd/m^2^) in luminance conditions. Following the adapter prestimulus period, the oriented (45° or 135° clockwise tilt) flickering gratings were presented for 4 s. Tritan gratings were created by adding the blue RGB channel (190, 190, 230 RBG; 58.86 cd/m^2^) to defined alternating regions of a central aperture in the yellow adapter (see Fig. 1). The resulting stimulus appeared to observers as a white and yellow grating with a visual angle of 3.10° and was flickered at 7.5 Hz (driving frequency), exhibiting a spatial frequency of 2.90 cycles/°. Luminance gratings were created by adding white (255, 255, 255 RBG; 78.95 cd/m^2^) to defined alternating regions of a central aperture in the black adapter and exhibited the same visual angle, flicker frequency, and spatial frequency as the tritan gratings (see Fig. 1).

The bright yellow adapter prestimulus period functioned to minimize the luminance sensitivity of M- and L-cones, so that slight changes in luminance (tritan grating presentation) may not surpass their luminance contrast threshold (10, 11, 43). As a result, the tritan gratings appeared near-equiluminous, and the opponent signals conveyed by the tritan grating should predominantly be transmitted through S-cones. The Michelson contrast of the tritan gratings was 3% and 99% for the luminance gratings. Verification of stimulus calibration was measured using a Gossen MavoSpot Luminance meter. In addition, the flicker frequency is an important consideration when examining S-cone-mediated responses in the visual cortex. Previous work has shown evidence of the attenuation of S-cone sensitivity as the temporal frequency of visual stimulation increases (44, 45). Importantly, stimulation frequencies beyond 10 Hz may not be suitable for evoking S-cone activation (44, 45). For instance, some studies have observed peak S-cone-mediated responses to flicker frequencies of 4 and 8 Hz in early visual cortex (14, 46). Therefore, the use of a relatively moderate flicker frequency (7.5 Hz) enabled the examination of S-cone-mediated conditioning effects in the visual cortex.

The unconditioned stimulus (US) was a mild electrical pulse administered using a Digitimer DS7A constant current stimulator (Hertfordshire, UK) connected to an electrode placed on the participant’s nondominant wrist. The pulse duration was 150 ms and co-terminated with the flickering grating in trials where the orientation of the grating was paired with the mild electrical stimulus.

#### Procedure.

Participants were seated ~120 cm in front of the stimulus presentation monitor in an electrically shielded, sound-attenuated room with dim lighting. Once the electroencephogram (EEG) sensor net was applied, the pupillometry system and electrical stimulus intensity were adjusted and individually set for each participant. Before beginning the experiment, participants were provided with written and verbal instructions describing that they would see a series of flickering patterns on the screen and would occasionally feel an annoying electrical stimulus. Participants completed expectancy ratings throughout the task and were asked to rate the likelihood that they would expect to feel the annoying electrical stimulus when shown each flickering pattern. The expectancy ratings involved dragging the mouse pointer across a scale where the far left of the scale indicated “not likely that the flickering pattern was accompanied by a shock,” the middle indicated “uncertain,” and the far right indicated “certain that the flickering pattern was accompanied by a shock.”

The intensity of the electrical stimulus was determined during the shock work-up phase and was set individually for each participant before beginning the task. The shock work-up consisted of repeated electrical stimulus pulses that started at 0 mA and increased incrementally by 0.1 mA. After each electrical stimulus pulse, the participants rated the intensity of the stimulus using a pain scale where 0 indicated that the stimulus was not annoying, aversive, or painful and 10 indicated that the stimulus was extremely painful. The intensity was increased until the participants rated the electrical stimulus as a level 4 on the pain scale, indicating that the stimulus was highly aversive and uncomfortable, but not painful. The shock work-up consisted of two separate work-ups to a level 4 on the pain scale. The intensity level was recorded after each work-up, averaged together, and then an additional 25% of the average intensity level was added to reach the final intensity used throughout the experiment. The final intensity level was delivered to the participant immediately before beginning the experiment to ensure that it was still tolerable and not painful. If the final intensity was reported to be painful and no longer tolerable, the intensity was reduced to the mean amperage prior to the 25% increase. However, if the intensity was still intolerable, then the intensity was reduced until it was tolerable for that participant. The average electrical stimulus intensity was 0.87 ± 0.68 mA (*M* ± SD).

The task consisted of 160 trials, with 20 trials in the habituation and extinction phases and 120 trials in the acquisition phase. During the habituation phase, participants were introduced to each of the stimulus types without the presence of the mild electrical stimulus (US) and prompted to provide expectancy ratings after completing the 20 trials. In the acquisition phase, the US was introduced, and the orientation (45° or 135° clockwise tilt) of the grating signaled the presence (CS+) or absence (CS−) of the mild electrical stimulus in both the tritan and luminance conditions (see Fig. 1C). Participants provided expectancy ratings after the first 20 trials of the acquisition phase and then again after 80 trials. The orientation of the CS+ grating was counterbalanced across participants, and the reinforcement rate during the acquisition phase was 100%. The extinction phase was included primarily for ethical reasons to reduce the association between the CS+ and the US. Participants also completed expectancy ratings after the 20 trials of extinction. Throughout the task, the occurrence of a given trial type was equiprobable and pseudorandomized.

### Data Acquisition and Processing

#### Pupillometry.

Pupil data were recorded with an EyeLink 1000 Plus system on a 16-mm lens (SR Research) and sampled at 500 Hz. The data were filtered offline using a Butterworth sixth-order low-pass filter with a cutoff frequency of 0.03 Hz. Next, segments of 600-ms prestimulus onset and 3,800-ms poststimulus onset were extracted from the continuous data, and segments with missing pupil data were identified and estimated using cubic interpolation. The resulting segments were averaged across conditions and baseline corrected to the average pupil diameter of the 200 ms prior to stimulus onset. One participant did not have pupil data and was excluded from subsequent analyses.

### Electroencephalography

Continuous EEG data were recorded using a 129-electrode high-density, high-impedance EEG System (EGI) with Cz as the recording reference and sampled at 500 Hz. Offline data processing was completed using a custom-automated EEGLAB pipeline in MATLAB (47), and the code can be found here https://osf.io/yue78/. Continuous EEG data were filtered using a Butterworth fourth-order bandpass filter with a lower and upper cutoff frequency of 3 and 25 Hz, respectively. Eye blinks were corrected using the automated eye correction procedure (48) in the BioSig open-source package for biomedical signal processing (49). Next, epochs of 600-ms prestimulus to 3,800-ms poststimulus onset were extracted from the continuous EEG data. For both the luminance and tritan conditions, CS− and CS+ trials were segmented to exclude the onset of the US to preserve trials in the CS+ conditions that can be disproportionally affected by movement artifacts produced in anticipation of the US onset.

Artifact correction and rejection were carried out according to the Statistical Control of Artifacts in Dense Sensors Arrays (SCADS) procedure (50). The SCADS approach computed a compound quality index for each participant, channel, and trial using three statistical parameters (50): rectified (absolute) mean amplitude, standard deviation, and maximum transient voltage change. The compound quality index was evaluated for each channel, and channels with high artifact content throughout the recording (globally bad channels) were flagged when the quality index exceeded 2.5 standard deviations above the median of the quality index. Globally bad channels were interpolated using a 2-D spline interpolation method, where all channels that survived artifact correction and rejection were used to estimate the values of the flagged channel. Channels at the trial level were evaluated using the same threshold for the quality index, and flagged channels were interpolated within that trial using the same spline method. Finally, trials were flagged for rejection if the compound quality index exceeded 1.25 times the median of the quality index. An average of 4% of channels in the acquisition phase were interpolated across participants (min: 0 channels; max: 15 channels), and an average of 91.60% of the total trials in the acquisition phase were retained for further analysis (tritan CS+ = 27.64; tritan CS− = 26.94; luminance CS+ = 27.86; luminance CS− = 27.48 trials on average out of 30).

The preprocessed and trial-averaged EEG data were prepared for the Hilbert transform to extract the time course of the ssVEP amplitude envelope for each condition (51). To this end, the EEG data were averaged across conditions for individual subjects, average referenced, and baseline corrected to the average voltage of the 200 ms prior to stimulus onset. Next, the baseline corrected data were filtered using two ninth-order Butterworth filters with an upper cutoff frequency of 8 Hz and the other with a lower cutoff frequency of 7 Hz. The narrow filtering extracted the frequency that the gratings flickered at 7.5 Hz, so the results could be interpreted in a frequency band-specific manner (52). The filtered data were Hilbert transformed using the MATLAB hilbert.m function to extract the time-varying estimates of phase and amplitude from the analytic signal over the entire epoch (−600 ms to 3,800 ms), in the freqtag_hilbert.m function from the FreqTag MATLAB toolbox (51). The Hilbert transform computes the analytic signal by doubling the positive-frequency Fourier coefficients, zeroing the negative-frequency coefficients, and taking the inverse of the Fourier transform of the doubled and zeroed Fourier coefficients (52). In the time domain, the analytic signal is a phase-shifted version of the original narrowly bandpassed time series.

### Statistical Analyses

#### Expectancy ratings.

US expectancy ratings were analyzed using paired *t* tests conducted in JASP (53). The expectancy ratings after the habituation phase were examined to verify that there were no stimulus-driven differences in expectancy in the absence of the US. Moreover, expectancy ratings during acquisition were analyzed to validate the aversive conditioning design, and expectancy ratings for the extinction phase were included for ethical reasons.

#### Steady-state visual evoked potentials.

The ssVEP responses were analyzed within conditions (tritan and luminance) using a nonparametric Bayesian bootstrap approach (40, 41) to estimate support for the hypothesis that the ssVEP amplitude at a given time point and occipital sensor was larger in response to the conditioned threat (CS+) stimuli compared to the conditioned safety (CS−) stimuli. The Bayesian bootstrap approach is rooted in methods developed to examine statistical effects across high-dimensional neural data (54). This mass-univariate approach enabled the investigation of conditioning effects without the need for rigid a priori regions or time windows. However, this approach is at risk of resulting in false positives due to the very large number of comparisons. To address this problem, the present study applied several correction procedures in addition to leveraging properties of the Bayesian approach (54). The Bayesian bootstrap procedure is a conservative data-driven method that quantifies the evidence of support for the hypothesis that at any time point and occipital sensor, visuocortical activation in response to the CS+ stimuli will be larger compared to the response to the CS− stimuli, separately for the luminance and tritan conditions. Because Bayes factors express support for a given hypothesis rather than the probability of a given finding under the null hypothesis, they are already less susceptible to multiple comparison problems (55, 56). To further control for multiple comparisons however, and to emphasize the most robust differences, widely accepted conservative thresholds for Bayesian support (57) were used to interpret effects: specifically, we interpreted only sensors and peak timepoints exhibiting decisive support (logarithmic Bayes factors, logBF10 of >2, i.e., the evidence for a difference is 100 times stronger than the evidence for the absence of a difference). To further improve the sensitivity of the Bayesian bootstrap analysis (58), only condition differences at occipital sensors were examined because the ssVEP is generated in the early visual cortex (59).

As suggested by Efron (41), evidence supporting the hypothesis that ssVEP amplitude was greater in response to CS+ compared to CS− trials was quantified by bootstrapping the condition differences (2,000 iterations) as well as a null distribution (calculated through permuting condition labels within subjects, 2,000 iterations). Bootstrapped distributions were generated by randomly drawing a participant’s amplitude difference with replacement, for each iteration (40). The null distribution was generated by randomly drawing a participant’s amplitude difference after randomizing the conditions with replacement, for each iteration. Before computing the Bayes factors, distributions of condition differences and null distributions were fit to a normal distribution after joint z-normalization to maintain the mean and spread of the distributions. Logarithmic Bayes factors (log10BF) were computed as the posterior odds that the distribution of condition differences was outside the null distribution (increased separation between the condition differences and null distribution), relative to uninformative (flat) prior odds of 1. Greater evidence to support the null hypothesis (BF01; increased overlap between the condition differences and null distributions) would have suggested that there were no ssVEP amplitude differences between the CS+ and CS− stimuli. Bayes factors were interpreted using Jeffreys’ scale (57), where values ranging from 0.5 to 1 indicated substantial support, 1 to 1.5 indicated strong support, 1.5 to 2 indicated very strong support, and values greater than 2 indicated decisive support for our hypothesis. Finally, the magnitudes of the conditioning effects were examined across luminance and tritan conditions by computing the ratio of Bayes factors (transitive Bayes factors) in the tritan condition to the Bayes factors in the luminance condition. Transitive Bayesian comparisons were carried out to explore the relative degree to which the conditioning effects observed within conditions remained robust (logBF10 > 2) when comparing across conditions.

#### Pupillometry.

Finally, pupil dilation was analyzed using a Bayesian repeated-measures ANOVA in JASP (53) to test the hypothesis that CS+ trials would evoke greater pupil re-dilation compared to CS− trials, regardless of condition.

## RESULTS

### US Expectancy Ratings

**Figure 2.**
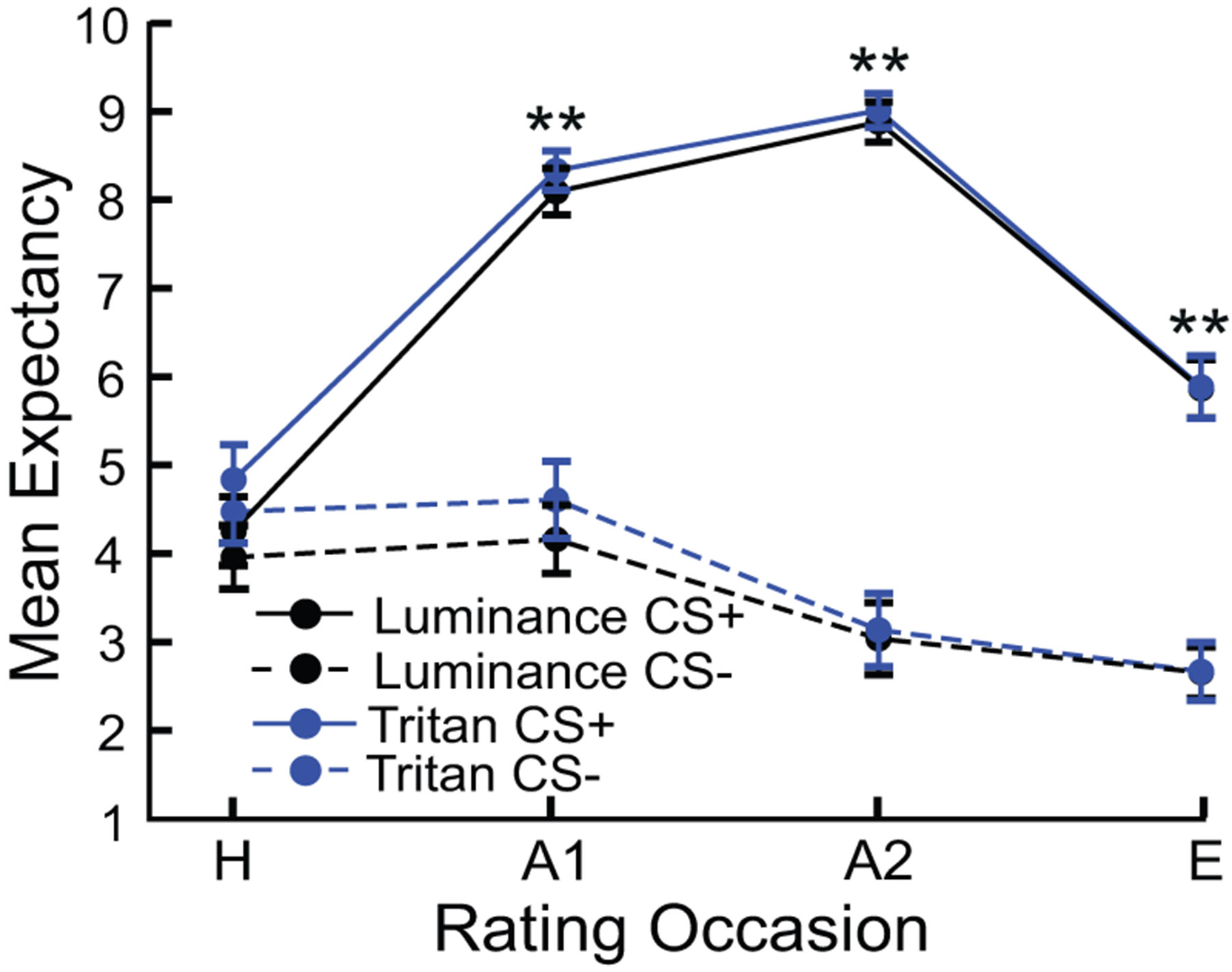
Mean unconditioned stimulus (US) expectancy ratings where 0 indicated low expectancy that the flickering grating was accompanied by a shock, 5 indicated uncertainty, and 10 indicated high expectancy that the flickering grating was accompanied by a shock. The habituation and extinction phases included one rating occasion (H and E), and the acquisition phase included two rating occasions (A1, 20 trials into acquisition; A2, 80 trials into acquisition). Paired *t* tests indicated that US expectancy ratings for the CS+ stimuli were significantly higher (**, *n* = 50 subjects; *P* < 0.001) compared to the ratings for the CS− stimuli during the acquisition and extinction rating occasions, regardless of condition.

Figure 2 shows the mean expectancy for the CS+ and CS− stimuli at each rating occasion (habituation, H; acquisition 1, A1; acquisition 2, A2; extinction, E) in the tritan and luminance conditions. The expectancy ratings were compared across stimuli at each rating occasion and for each condition using paired samples *t* tests computed in JASP (53). US expectancy analysis revealed that there were no significant differences in expectancy across stimulus type for the habituation rating occasion in the luminance [*t*(49) = 1.17, *P* = 0.250, *d* = 0.17] and tritan conditions [*t*(49) = 1.59, *P* = 0.119, *d* = 0.23]. However, for each acquisition rating occasion, the CS+ stimuli elicited significantly higher expectancy compared to the CS− stimuli for both the luminance [A1: *t*(49) = 7.11, *P* < 0.001, *d* = 1.01; A2: *t*(49) = 11.06, *P* < 0.001, *d* = 1.56] and tritan conditions [A1: *t*(49) = 6.63, *P* < 0.001, *d* = 0.94; A2: *t*(49) = 11.13, *P* < 0.001, *d* = 1.57]. Moreover, expectancy ratings were significantly higher for CS+ stimuli compared to CS− stimuli for the extinction rating occasion in the luminance [*t*(49) = 7.67, *P* < 0.001, *d* = 1.08] and tritan conditions [*t*(49) = 7.94, *P* < 0.001, *d* = 1.12]. Therefore, fear conditioning was successful across both experimental conditions.

### Steady-State Visual Evoked Potentials

**Figure 3.**
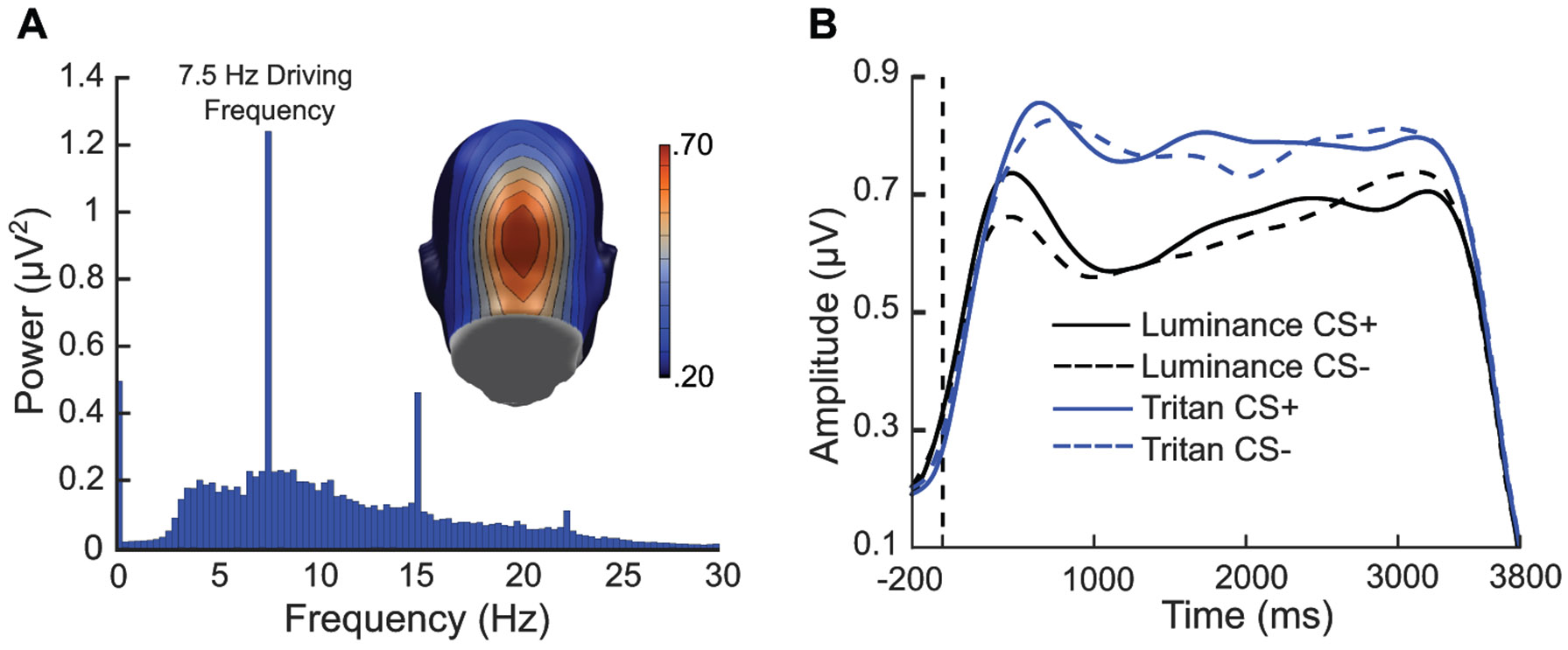
The Fourier and Hilbert transformed steady-state visual evoked potential (ssVEP) results (*n* = 50 subjects). *A*: the frequency spectrum of the ssVEP response to the tritan CS+ stimulus collapsed across participants at sensor Oz. The spike in the frequency spectrum at 7.5 Hz indicates the presence of the driving frequency. *B*: the results of the Hilbert transform at sensor Oz for all conditions and stimulus types collapsed across participants.

Figure 3A shows the frequency spectrum of the visuocortical activity at sensor Oz (60) in the tritan CS+ condition across all participants in the acquisition phase. The spike in the frequency spectrum at 7.5 Hz indicates that the driving frequency was present in the spectrum. Furthermore, Fig. 3B shows the results of the Hilbert transform at sensor Oz (60) across participants for each trial type in each condition, indicating that the driving frequency elicited visuocortical amplification regardless of condition.

**Figure 4.**
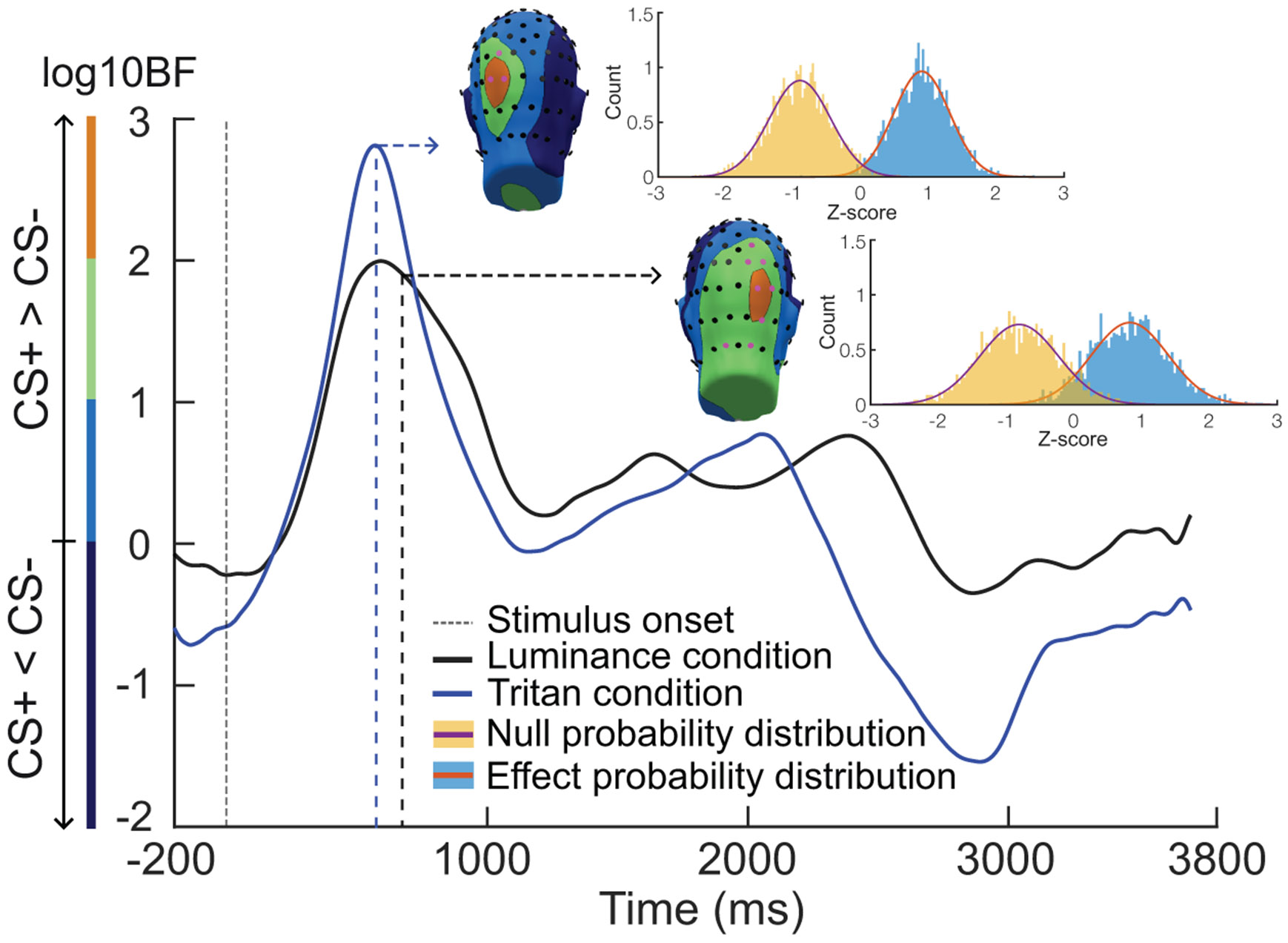
The ssVEP responses were analyzed within conditions (tritan and luminance) using a nonparametric Bayesian bootstrap approach. The condition level average log base 10 Bayes factor (log10BF; *n* = 50 subjects) time course across all occipital sensors exhibiting decisive support for the presence of a conditioning effect (log10BF > 2; magenta sensors) is shown separately for each condition. Topographies were plotted at the peak time point, averaged across sensors included in the time course. Density plots show the Bayesian bootstrapped z-transformed null and conditioning effect distributions averaged across sensors included in the time course and plotted at the average peak time point, separately for each condition. Distributions included 2,000 draws. Note that the distributions were z-transformed together to facilitate distribution fitting, so the null distributions are not centered around zero.

Figure 4 shows the results of the nonparametric Bayesian bootstrap approach used to estimate support for the hypothesis that the ssVEP amplitude at a given time point and occipital sensor was larger in response to the CS+ stimuli compared to the CS− stimuli, separately for each condition. Log base 10 Bayes factors (logBF10) were interpreted using Jeffreys’ scale (57) where values ranging from 0.5 to 1 indicated substantial support, 1 to 1.5 indicated strong support, 1.5 to 2 indicated very strong support, and values greater than 2 indicated decisive support for the presence of a conditioning effect. For the luminance condition, decisive evidence (log10BF > 2) of a conditioning effect was shown in eight right-lateralized occipital sensors [73, 76, 77, 83 (O2), 84, 88, 89, 90]. In six of the eight sensors that exhibited decisive support, peak logBF10 values occurred between ~500 and 700 ms after grating onset [73, peak log10BF = 2.10 at 618 ms; 76, peak log10BF = 2.22 at 506 ms; 77, peak log10BF = 2.14 at 492 ms; 83 (O2), peak log10BF = 2.18 at 692 ms; 84, peak log10BF = 2.24 at 576 ms; 90, peak log10BF = 2.35 at 622 ms] and in the remaining two sensors peak logBF10 values occurred around 900 ms after grating onset (88, peak log10BF = 2.19 at 910 ms; 89, peak log10BF = 2.27 at 914 ms). For the tritan condition, decisive evidence of a conditioning effect was shown in three right-lateralized occipital sensors [65 (O1), 66, 70]. Peak logBF10 values occurred between ~525 and 600 ms after grating onset [65 (O1), peak log10BF = 3.53 at 592 ms; 66, peak log10BF = 2.62 at 592 ms; 70, peak log10BF = 2.49 at 526 ms]. In addition, Fig. 4 shows the bootstrapped joint z-normalized effect and null distributions averaged across sensors that exhibited decisive evidence of an effect and plotted at the average peak time point corresponding to the peak log10BF value identified for each sensor, separately for the luminance (666 ms) and tritan (570 ms) conditions. The luminance distributions showed less overlap between the null and effect distributions, compared to the tritan distributions.

**Figure 5.**
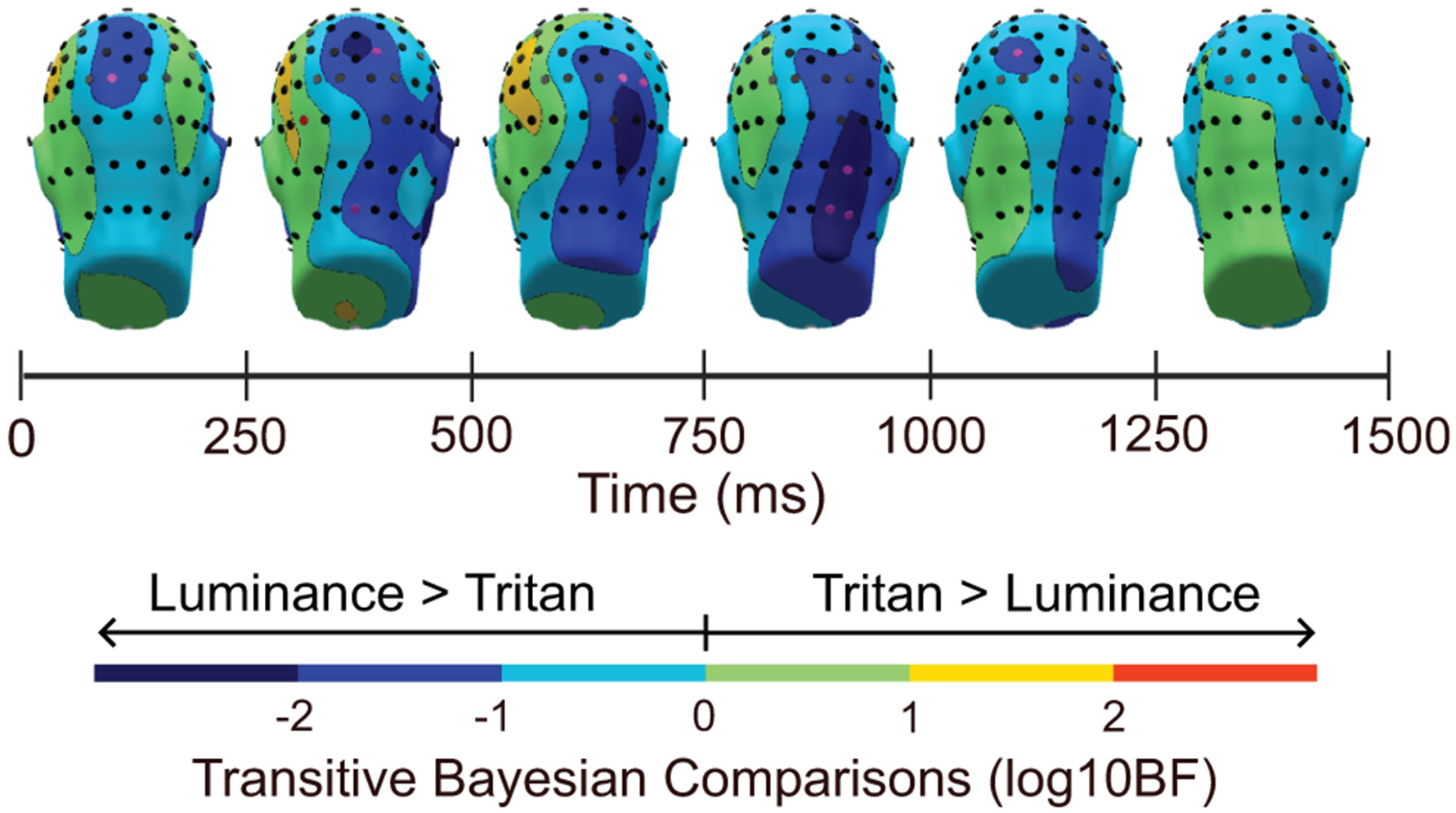
Topographic map of transitive Bayes factors computed as the ratio of the Bayes factors for the tritan conditioning effect to the Bayes factors for the luminance conditioning effect (*n* = 50 subjects). Negative transitive Bayes factors indicated that the luminance conditioning effect was larger than the tritan conditioning effect, and positive transitive Bayes factors indicated that the tritan conditioning effect was larger than the luminance conditioning effect. Transitive Bayes factor values greater than ∣2∣ indicated decisive support favoring one conditioning effect over the other.

Figure 5 shows the topographical map of transitive Bayes factors (logBF10). Transitive Bayes factors were computed as the ratio of Bayes factors in the tritan condition to the Bayes factors in the luminance condition. Negative transitive Bayes factors indicated increased conditioning effect magnitude in the luminance condition compared to the tritan condition, and positive transitive Bayes factors indicated increased conditioning effect magnitude in the tritan condition compared to the luminance condition at a given time point and occipital sensor. Transitive Bayes factors showed decisive evidence (log10BF > −2) of a larger luminance conditioning effect in a considerable number of central and right-lateralized occipital sensors [67, 71, 77, 81, 83 (O2), 84, 88, 89, 90, 94]. Peak logBF10 values greater than −2 occurred between ~200 and 1,000 ms after grating onset [67, peak log10BF = −2.04 at 1,032 ms; 71, peak log10BF = −2.06 at 190 ms; 77, peak log10BF = −2.12 at 388 ms; 81, peak log10BF = −2.11 at 464 ms; 83 (O2), peak log10BF = −2.15 at 758 ms; 84, peak log10BF = −2.24 at 544 ms; 88, peak log10BF = −2.87 at 932 ms; 89, peak log10BF = −2.80 at 944 ms; 90, peak log10BF = −3.06 at 576 ms; 94, peak log10BF = −2.49 at 928 ms]. The tritan conditioning effect was larger in one left-lateralized occipital sensor at 390 ms after grating onset (65, peak log10BF = 2.50 at 390 ms). Importantly, transitive Bayes factors were computed across conditions with different levels of adaptation (luminance and tritan).

### Pupil

**Figure 6.**
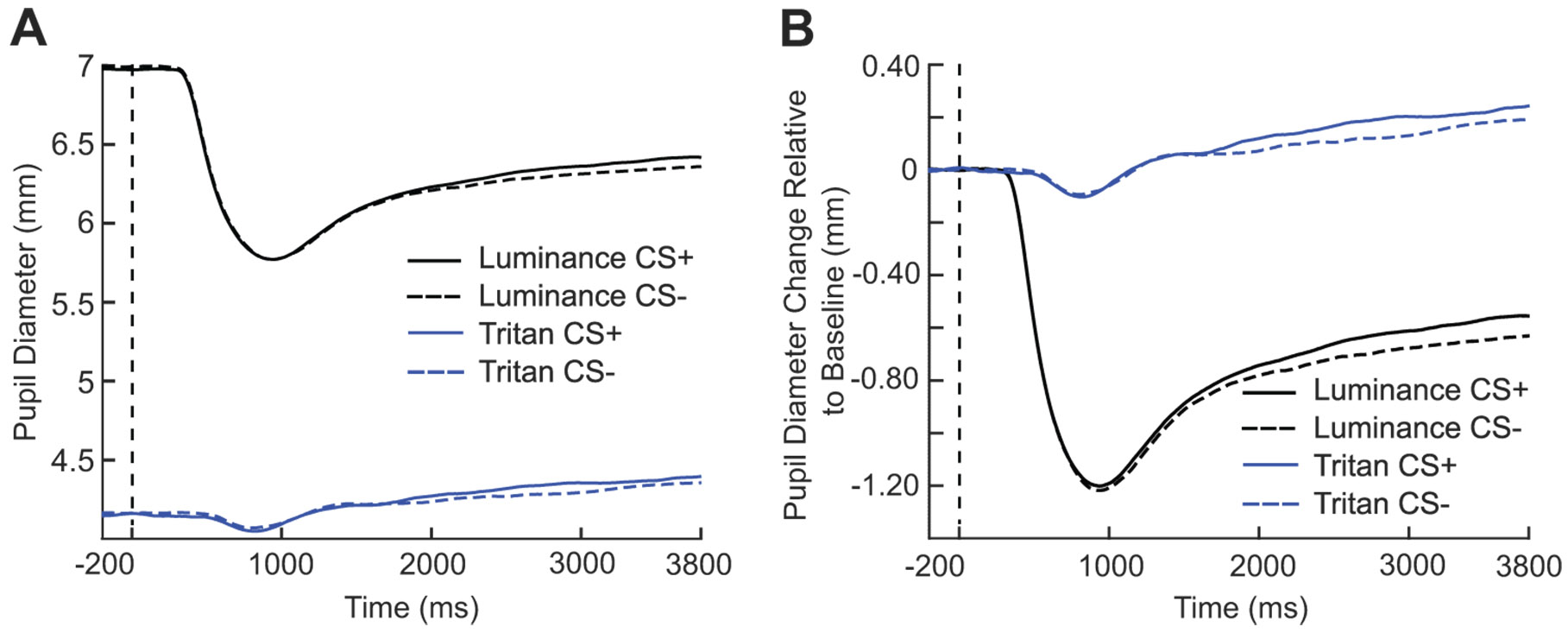
*A*: pupil diameter across conditions and stimulus types. *B*: changes in pupil diameter over time for each condition and stimulus type collapsed across all participants. The measurement window included in the 2 (Condition) × 2 (Stimulus) Bayesian repeated-measures ANOVA was 2,800 ms to 3,800 ms to assess differences in pupil re-dilation (*n* = 49 subjects). Bayesian post hoc tests showed decisive and very strong evidence of differences in pupil diameter across conditions (log10BF = 63.83) and stimulus types (log10BF = 1.58), respectively.

Figure 6 shows the results of the 2 (Condition) × 2 (Stimulus type) Bayesian repeated-measures ANOVA computed in JASP (53) that examined the effect of condition (luminance and tritan) and stimulus type (CS+ and CS−) on change in pupil diameter (mm) over the last two seconds of the epoch (1,800 ms–3,800 ms). The results indicated decisive support (log10BF = 32.49) for the model that included main effects of Condition and Stimulus type, and there was no evidence of an interaction. Bayesian post hoc tests showed decisive and very strong evidence of differences in pupil diameter across conditions (log10BF = 63.83) and stimulus types (log10BF = 1.58), respectively. Pupil diameter, regardless of stimulus type, was larger in the luminance condition (CS+, *M* = 6.32 mm; CS−, *M* = 6.28 mm) compared to the tritan condition (CS+, *M* = 4.33 mm; CS−, *M* = 4.29 mm), and CS+ stimuli elicited greater pupil re-dilation compared to the CS− stimuli, regardless of condition.

## DISCUSSION

The present study investigated the contribution of the koniocellular visual pathway to aversive learning in human visual cortex using the ssVEP technique to quantify electrocortical activation in response to conditioned threat and safety cues. We tested the hypothesis that differential aversive conditioning would enhance visuocortical activation to conditioned threat stimuli in the tritan condition, paralleling established findings for stimuli in which the full luminance information is available. S-cone biasing and achromatic stimuli were used to convey color-opponent and achromatic visual information to regions along the koniocellular pathway and luminance channels, respectively. Aversive learning within each condition was examined by comparing the ssVEP responses to conditioned threat (CS+) and safety (CS−) cues, and showed decisive evidence of increased visuocortical activation in response to CS+ stimuli compared to CS− stimuli in both the luminance and tritan conditions. For the tritan condition, decisive evidence of aversive learning was observed in several left-lateralized occipital sensors exhibiting peak log10BF values (>2) around 525–600 ms after stimulus onset. For the luminance condition, decisive evidence of aversive learning was found across several central and right-lateralized occipital sensors exhibiting peak log10BF values (>2) occurring around 500–700 ms and around 900 ms in two remaining right-lateralized occipital sensors. Consistent with the ssVEP results, pupil re-dilation and behavioral expectancy ratings showed evidence of increased sympathetic activation (39) and threat expectancy in response to the CS+ compared to the CS− stimuli in both conditions, respectively.

In line with our first hypothesis, tritan stimuli elicited robust ssVEP responses overall, extending previous findings of visuocortical amplification of S-cone biasing stimuli (17-21). Consistent with our second hypothesis, we observed greater visuocortical activation in response to the CS+ stimuli, compared to the CS− stimuli, in both the tritan and luminance conditions. Decisive support was shown for the conditioning effects in each condition (logBF10 > 2), suggesting that activity along the koniocellular pathway participates in the cortical representation of conditioned threat in humans. Although the present study provides evidence of the modulation of S-cone-driven signals by task cues in primary visual cortex, these results have not been previously observed in attention paradigms utilized by Wang and Wade (21) and Highsmith and Crognale (20). However, the results are generally consistent with previous findings from Martinovic et al. (23), who observed attentional modulation of the N1 early sensory visual-evoked potential driven by S-cone biasing stimuli. The N1 component was modulated by task cues and showed increased discrimination between cued and uncued visual inputs.

Moreover, our findings aligned with differential aversive conditioning studies that used luminance channel biasing stimuli and observed robust visuocortical amplification of conditioned threat cues compared to safety cues over central and right-lateralized occipital sensors (61, 62). Differences in the topographies of the conditioning effect in the luminance and the tritan conditions could be related to the differences in stimulus chromaticity. It has been established that regions along the ventral visual stream are sensitive to color, and achromatic visual information largely activates regions in the dorsal stream (63, 64), which could begin to explain the differences in effect lateralization across conditions. However, future investigations using neuroimaging methods with better spatial resolution are needed to address the involvement of different extrastriate regions. Overall, the topographies of the conditioning effects were consistent with findings showing early visual area contribution to the generation of ssVEP responses (59).

Early visual cortex contributes to the amplification of aversive stimuli through feedforward projections from V1, local inhibitory circuits, and feedback projections from high-order visual areas (9, 65-67). Therefore, the differences in the temporal characteristics and spatial emergence of robust conditioning effects in both the luminance and tritan conditions could be attributed to the specific neurophysiological connections associated with each channel. The input layer of V1 (Layer 4C) receives substantial excitatory geniculo-cortical innervation, including magnocellular and parvocellular geniculo-cortical projections (68-71). Conversely, the koniocellular pathway exhibits distributed geniculo-cortical projections to the output layers of V1, including layer 1 and the cytochrome oxidase blobs in layer 2/3 in primates (25, 69, 72).

It has been hypothesized that the laminar layers of V1 are functionally distinct, and several studies have modeled how the functional property of orientation selectivity emerges in V1 across the laminar layers (65, 73, 74). Ringach et al. (75) provided evidence of neurons in V1 that displayed diverse orientation selectivity and contributed to broadly distributed orientation selectivity across the layers of V1 in primates. Moreover, Gur et al. (73) found that the distribution of orientation tuning in V1 in primates is not uniform, and differences in tuning could be attributed to the variability of excitatory and inhibitory mechanisms in each layer of V1. For instance, local inhibition is thought to sharpen the broadly tuned excitatory projections from the LGN and may facilitate the first stage of steady-state orientation-specific responses observed over early visual areas (65, 66). Moreover, feedback projections from higher-order visual areas are also thought to play a role in modulating the magnitude of the ssVEP by conveying information about arousal to early visual areas and displaying a pattern of retinotopic alignment with feedforward projections from V1 in primates (60, 67, 76, 77). Therefore, differences in higher-order feedback projections and local inhibitory interactions that shape the ssVEP responses could contribute to the robust conditioning effects observed in both the luminance and tritan conditions. Further investigation is needed to disentangle the precise temporal boundaries of these effects across the luminance and tritan conditions.

To further elucidate the koniocellular contribution to aversive learning, transitive Bayes factors were computed to compare the relative magnitude of the conditioning effects across conditions. Luminance-mediated conditioning effects were found to be larger compared to S-cone mediated effects in several central and right-lateralized occipital sensors that showed peak log10BF values (>2) occurring around 200–1,000 ms after stimulus onset. Although these results suggest that S-cone signals may be less sensitive to differential aversive conditioning, differences in adaptation across conditions cannot be ruled out. Future directions include examining conditioning effects across conditions where similar levels of adaptation have been applied to ensure that the observed differences are driven by the properties of the early visual pathways, and not adaptation. Furthermore, the current study focuses on conditioning effects present in the fundamental frequency, adding to an existing literature on aversive conditioning using this same approach. However, luminance channels tend to exhibit more transient responses (magnitude is distributed across the higher harmonics) when compared to chromatic responses (78-81), so we have included a supplementary analysis of the average visuocortical response magnitude in each condition across the fundamental frequency and second harmonic (see Supplemental Fig. S1).

Although the tritanopic technique functions to mitigate minor luminance contributions from S-cone signals under chromatic adaptation (10) and has been used in studies with broadly similar goals (11), we conducted a control analysis to examine potential luminance artifacts in the visuocortical responses to the tritan stimulus. The control experiment included a “no-blue tritan” stimulus (yellow grating on top of the original yellow background) with matched luminance and Michelson contrast to the tritan stimulus. The no-blue tritan stimulus facilitated the comparison between visuocortical activity evoked by blue-on yellow-off color-opponent stimulus properties and a control stimulus lacking color-opponent properties known to bias response properties of the koniocellular pathway. Similarly, an achromatic condition with the same luminance and contrast as the no-blue tritan stimulus was also included. All other aspects of the task remained the same. Data were collected from four participants and processed using identical collection and processing procedures as the main study. Results indicated strong attenuation of the visuocortical response to the no-blue tritan in both the CS− and CS+ stimulus conditions when compared to the tritan CS− and tritan CS+. In fact, both the no-blue tritan and the achromatic stimuli resulted in attenuated visuocortical activation, suggesting that the 3% luminance contrast does contribute to the ssVEP signal in the present study, but only weakly, with the majority of the signal likely stemming from other channels. Supplemental Fig. S2 shows the average visuocortical activation for each of the control stimuli at the fundamental frequency and the second harmonic.

The present study extends our knowledge of the functional properties of the koniocellular visual pathway. These results provide robust evidence of the koniocellular involvement in aversive learning in human visual cortex, suggesting that the koniocellular pathway is sensitive to aversive conditioning. In addition, comparisons across conditioning effects showed increased visuocortical evidence of aversive learning in responses to stimuli that engage luminance channels, compared to stimuli that activate the response properties of the koniocellular visual pathway. Future investigations are warranted to determine whether differences in adaptation across conditions contributed to the enhanced sensitivity of the luminance channels to aversive conditioning. Importantly, the present study demonstrated that aversive conditioning modulated both koniocellular and luminance channel signals in the human visual cortex.

## Supplementary Material

Supplemental Fig. S1: https://osf.io/stcpg/files/ewu3q.

Supplemental Fig. S2: https://osf.io/stcpg/files/z5qa9.

## Data Availability

Source data for this study are openly available at https://osf.io/yue78/overview. Source data includes, all preprocessed electrophysiological data, pupil data, and limited demographic data. Data processing code and analysis code are also openly accessible at https://osf.io/yue78/overview.
